# Comprehensive Analysis of Chlorine-Induced Aging in High-Density Polyethylene: Insights into Structural, Thermal, and Mechanical Degradation Mechanisms

**DOI:** 10.3390/polym18010014

**Published:** 2025-12-21

**Authors:** Elena-Emilia Sirbu, Maria Tănase, Alin Diniță, Cătălina Călin, Gheorghe Brănoiu, Ionuț Banu

**Affiliations:** 1 Chemistry Department, Petroleum-Gas University of Ploiești, 100680 Ploiesti, Romania; elena.oprescu@upg-ploiesti.ro; 2 Mechanical Engineering Department, Petroleum-Gas University of Ploiești, 100680 Ploiesti, Romania; adinita@upg-ploiesti.ro; 3 Petroleum Geology and Reservoir Engineering Department, Petroleum-Gas University of Ploiești, 100680 Ploiesti, Romania; gheorghe.branoiu@upg-ploiesti.ro; 4 Department of Chemical and Biochemical Engineering, National University of Science and Technology POLITEHNICA Bucharest, 060042 Bucharest, Romania; ionut.banu@upb.ro

**Keywords:** HDPE, aging, chlorine action, XRD, DSC, TGA, FTIR, mechanical performance

## Abstract

This study investigates chlorine-induced aging of high-density polyethylene (HDPE) through a 3 × 3 factorial matrix combining three temperatures (20, 40, 60 °C) and three chlorine concentrations (5, 10, 20 ppm) over 45 days. Tensile tests revealed progressive embrittlement, with elongation at break decreasing sharply under severe aging; samples exposed to 60 °C and 20 ppm exhibited premature brittle failure despite peak stresses remaining near ~22 MPa. XRD results showed a reduction in crystallinity from 67.07% (reference) to 61.06–61.31% under the most aggressive conditions, accompanied by a decrease in crystallite size from 5.60 nm to 2.10–2.50 nm. FTIR analysis confirmed oxidation through increased carbonyl absorption at 1716 cm^−1^ and new bands at 1608–1635 cm^−1^. TGA revealed substantial thermal deterioration, with T5% falling from 450 °C (reference) to 327 °C at 60 °C/20 ppm, along with an additional degradation peak at 398 °C. DSC showed a melting temperature decrease from 136.32 °C to 131.67 °C and an increase in crystallinity from 41.07% (unexposed sample) to 59.19% (60 °C/20 ppm). Statistical analysis of the results established that degradation is governed by different dominant factors depending on the measured property: Chlorine concentration was found to be the dominant factor for XRD crystallinity and thermal decomposition T5%, confirming that surface structural damage and early molecular weight loss are driven primarily by chlorine-induced oxidation. Conversely, DSC crystallinity was governed primarily by temperature, reflecting thermally driven molecular reorganization within the bulk material. Overall, chlorine exposure, amplified by temperature, accelerates chemical oxidation, structural degradation, and mechanical embrittlement, reducing the long-term reliability of HDPE in chlorinated water systems. The findings provide critical data for predicting the service life and informing material selection for HDPE components used in high-temperature or high-chlorine water distribution systems.

## 1. Introduction

High-Density Polyethylene (HDPE) remains a material of choice within global drinking water distribution systems, valued for its relatively low cost, favorable productivity, light weight, and perceived high resistance to standard chemical degradation [1,2,3].

These attributes have historically underpinned lifetime expectations often exceeding 50 years, positioning HDPE as a robust material for critical infrastructure [4,5]. However, this projected longevity is increasingly challenged by the continuous exposure of the material to chemical disinfectants, mandatory for maintaining potable water quality [6].

ClO_2_ is favored over other disinfectants—such as free chlorine (from sodium hypochlorite, calcium hypochlorite, or gaseous chlorine) and chloramine—because of its high oxidation potential and its ability to inactivate viruses and pathogens that are resistant to conventional chlorination [6].

The primary consequence of employing chemical disinfectants is the initiation of polymer degradation, which significantly shortens the material’s service life [6]. While the widespread use of polymers as HDPE continues, comparative studies demonstrate a crucial need for a detailed understanding: Barbarosa et al. [1] noted that alternative materials, such as Polyvinyl Chloride (PVC), exhibit more stable overall behavior when in continuous contact with chlorine solutions compared to HDPE and Polypropylene (PP). Given the immense financial and public health implications of premature pipeline failure, a comprehensive, multi-scale analysis that links the underlying chemical attack to the macroscopic mechanical failure is essential for accurate infrastructure management and lifetime prediction.

The maximum permitted concentration of ClO_2_ in tap water varies by country, for example: 0.8 mg/L in the USA, 0.25 mg/L in Belgium, and 0.15 mg/L in Switzerland [6].

Chemical degradation immediately triggers morphological changes within the polymer matrix. The consensus in the literature is that the oxidative attack is preferential, occurring primarily within the less ordered, more accessible amorphous regions of the polyethylene structure [7]. Conversely, the highly ordered crystalline component is preserved during aging [8,9]. This selective degradation leads to a measurable increase in mass crystallinity in the degraded surface layer, widely reported as a key indicator of polyethylene aging [10,11,12,13]. These morphological changes are traceable through thermal analysis. Hassinen et al. [7] utilized Differential Scanning Calorimetry (DSC) to quantify this structural damage, reporting that the melting peak temperature of degraded HDPE shifted 3 °C lower than the un-degraded material after 438 h of aging under accelerated conditions. This shift, coupled with the observed broadening of the melting peak, indicates differences in crystallite size or perfection, likely resulting from the removal of the critical amorphous tie molecules that link the crystalline domains [8].

The chemical and morphological transformation from a tough, ductile polymer into a stiff, brittle layer manifests as a catastrophic failure of engineering properties. Early assessments using static tensile strength tests often fail to indicate significant initial changes [1,2,14]. However, this masks the underlying chemical damage, which rapidly leads to severe ductility loss. The loss of the high-molecular-weight chains, confirmed by GPC, shows 20–50% MW loss [15], is particularly damaging because these macro-chains act as “tie molecules,” bridging adjacent crystalline lamellae and preventing localized crack opening. Their scission removes the material’s ability to uniformly distribute stress and accommodate large strains, transitioning the failure mode from ductile to brittle.

Definitive quantitative evidence of mechanical failure was provided by Castegnetti et al. [16], who studied HDPE exposed to chlorinated water under pressure. Their findings showed that the elongation at fracture dropped dramatically to only 50%, compared to the control samples, which maintained ductility at 700%. This rapid and extreme loss of strain-at-break capability marks the transition to mechanical embrittlement [15].

In pressurized applications, this embrittlement precipitates failure via Slow Crack Growth (SCG). Stress crack resistance (SCR) testing is therefore the most relevant metric for predicting long-term pipe performance. Lavoie et al. [17] utilized Notched Constant Tensile Load (NCTL) tests on degraded HDPE geomembranes and demonstrated that the chemical aging process acted as a catalyst for stress cracking, resulting in a reduction in stress crack resistance by 50% to 60%. Dear and Mason [18] confirmed this mechanism through field observations of failed PE pipes, revealing that failure typically occurs by slow crack growth (SCG) due to creep, but only after the initial inner layer has been chemically degraded by chain scission and oxidation. The degradation of tensile properties and SCR has been shown to occur shortly after immersion, with increasing free chlorine concentration accelerating these mechanical degradation rates [19].

Paired comparison tests [2] showed a significant reduction only in tensile strength at break for HDPE pipes aged in 250 ppm Cl_2_ at 37 °C. Differences in elongation at break and tensile strength at yield were insignificant, though aged samples exhibited high variability in elongation and a brittle fracture mode, contrasting with the ductile behavior of unaged HDPE.

The novelty of this research is significantly amplified by its rigorous, matrix-based experimental design. While previous studies often focus on single or highly accelerated conditions, this work uniquely employs a 9-point factorial aging matrix by systematically varying three temperature levels and three chlorine concentrations. This allows for the decoupling and quantification of the individual and synergistic effects of temperature and oxidant concentration, which is critical for accurate, real-world lifetime prediction of HDPE infrastructure. The comprehensive analysis, integrating TGA, DSC, FTIR, XRD, and mechanical testing across all nine conditions, establishes a direct, quantitative correlation between the chemical changes (oxidation/chain scission via FTIR), subsequent alterations in crystallinity and thermal stability (XRD, DSC), and the final loss of critical mechanical properties, providing an unprecedented, multi-parametric dataset for understanding chlorine-induced degradation kinetics. HDPE was selected as the model material due to its widespread use in water and chemical infrastructure and its known susceptibility to oxidative degradation. Its well-characterized thermal, mechanical, and chemical properties make it an ideal candidate for systematically investigating the combined effects of temperature and chlorine concentration on material longevity.

## 2. Materials and Methods

### 2.1. Design of Experiments

The experimental design was established to evaluate the effects of chlorine concentration and temperature on the aging behavior of high-density polyethylene (HDPE). The study aimed to identify the most influential factors affecting both the mechanical and structural properties of the material. A commercial type of high-density polyethylene (HDPE) pipe was bought from a local shop.

HDPE specimens were exposed to chlorinated aqueous environments under controlled conditions, with two main variables considered: temperature and chlorine concentration. Three temperature levels were selected (20 °C, 40 °C, and 60 °C) to simulate mild, moderate, and accelerated aging conditions. Simultaneously, three chlorine concentrations were applied (5 ppm, 10 ppm, and 20 ppm), representing typical to aggressive chlorination scenarios encountered in industrial and domestic water systems.

The three chlorine concentrations (5, 10, and 20 ppm) and three temperatures (20, 40, and 60 °C) were selected to cover both realistic service conditions and accelerated aging scenarios. The lower concentration of 5 ppm corresponds to the upper limit of free chlorine typically maintained in potable water distribution systems, as recommended by the World Health Organization [20], while the higher concentrations (10 and 20 ppm) were employed to accelerate the degradation process and allow kinetic evaluation within a practical experimental timeframe. Similarly, the temperature levels were chosen to represent ambient conditions (20 °C), moderately elevated operational temperatures (40 °C), and an accelerated aging condition (60 °C), reflecting accelerated aging conditions used in previous experimental investigations for HDPE degradation [5,6]. This factorial design enables the systematic decoupling and quantification of individual and synergistic effects of temperature and chlorine concentration on HDPE degradation.

A full factorial design (3 × 3), as shown in Table 1, was implemented to systematically study the combined influence of these parameters, resulting in nine distinct experimental conditions. Each condition was maintained for an exposure period of 45 days to ensure observable aging effects. All tests were conducted in duplicate to improve statistical accuracy and reproducibility.

The resulting data were processed using Minitab version 19 statistical software. Pareto charts and main effects plots were generated to visualize and quantify the relative impact of each factor and its interactions on the measured responses. The Pareto analysis identified the most significant variables influencing the aging behavior, while the main effect plots provided insight into the direction and magnitude of influence of temperature and chlorine concentration on HDPE degradation. These analyses supported the identification of critical degradation mechanisms and guided the interpretation of changes in the structural and mechanical performance of HDPE after exposure. These graphical analyses and the corresponding discussion are presented in detail in Section 3.5, supporting the identification of critical degradation mechanisms and guiding the interpretation of changes in the structural and mechanical performance of HDPE after exposure.

### 2.2. Aging Procedure

The aging tests (Table 2) were conducted by immersing HDPE specimens in chlorinated aqueous solutions prepared at the designated concentrations of 5 ppm, 10 ppm, and 20 ppm of free chlorine. The chlorinated environments were prepared using a 15% sodium hypochlorite (NaOCl) solution, diluted with deionized water to reach the desired chlorine concentrations.

**Figure 1:**
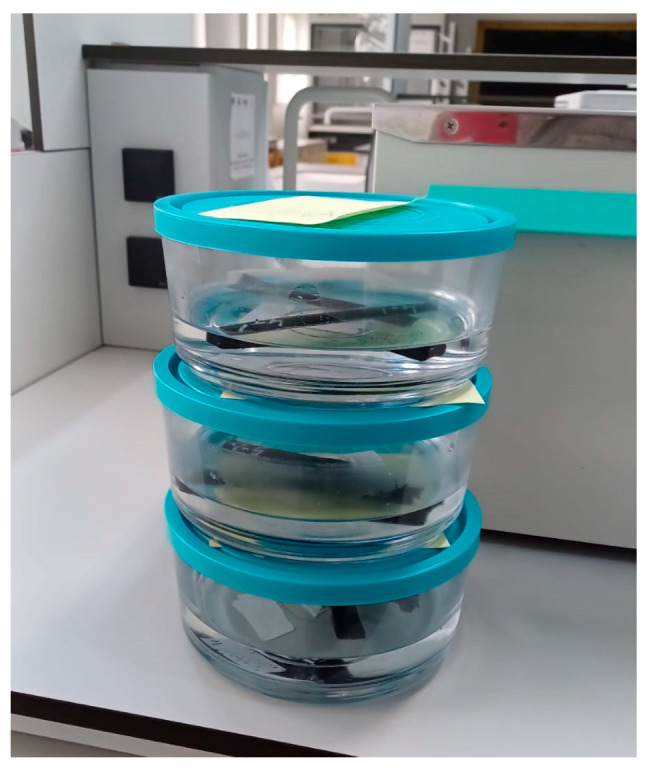
Specimens exposed to aggressive environment.

The test specimens were fully submerged in sealed glass containers to minimize chlorine volatilization and oxidation losses, as seen in Figure 1.

The tensile test specimens were prepared from a commercial PE100 high-density polyethylene (HDPE) pipe with a pressure rating of PN 10, corresponding to SDR 17, featuring an outer diameter of 90 mm and a wall thickness of 5.4 mm [21].

The fundamental material parameters of the HDPE samples prior to chlorine-induced aging are summarized in Table 3 [22].

### 2.3. Tensile Testing

Mechanical characterization of the HDPE specimens was performed after the 45-day aging period to evaluate the influence of chlorine concentration and temperature on the material’s performance.

Ten groups of specimens (including the control/unexposed samples—R), each consisting of three samples, were subjected to mechanical testing in accordance with the ASTM D638 standard [27] using the LRX Plus testing machine manufactured by Lloyd Instruments, with a maximum load capacity of 2.5 kN (LLOYD Instruments, Segensworth Fareham, UK), operating at a crosshead speed of 20 mm/min.

Before testing, samples were conditioned at room temperature for at least 24 h to achieve thermal and moisture equilibrium. The stress–strain data obtained from each test were used to highlight the influence of aggressive environments on HDPE pipes.

### 2.4. XRD Analysis

For the analysis of the samples by X-ray diffraction (XRD), a D8 Advance diffractometer (Bruker-AXS) was used, equipped with a copper anode X-ray tube (Cu-Kα radiation, λ = 1.54 Å), in a θ–θ configuration and Bragg–Brentano geometry. Measurements were performed using the XRD Commander v2.6.1 software over the 2θ range of 10–70°, with the following parameters: voltage 40 kV, anode current 40 mA, step size 0.1°, and scan rate 0.1°/5.

The identification of the polyethylene type was carried out using Diffracplus EVA v14 software and the PDF-ICDD database (ICDD card 00-054-1982). Quantitative determinations using the Rietveld refinement technique were performed with Diffracplus TOPAS 4.1, employing a pseudo-Voigt profile function for peak fitting.

The degree of crystallinity (X_C_) from the XRD spectrum was calculated using the following equation:

(1) $$X_{c}=\frac{\sum A_{cr}}{\sum A_{cr}+A_{am}}$$

where *A_cr_* is the fitted areas of the crystal phase, and *A_am_* is the fitted areas of the amorphous phase [28,29,30,31].

### 2.5. FT-IR Analysis

The functional groups of the samples that appeared after the thermochemical treatment were highlighted using a Shimadzu IR TRACER-100 FTIR spectrometer (Kyoto, Japan), recording in the scanning range of 4000–400 cm^−1^. The variation in functional groups was evaluated by calculating the carbonyl index by the ratio of the absorption of the carbonyl group around 1716 cm^−1^ to the reference peak at 1462 cm^−1^ [32].

### 2.6. Thermal Analysis

Thermogravimetric analyses were conducted using TGA/DTG equipment (TGA 2 Star System Mettler Toledo, Zurich, Switzerland), varying temperatures from 25 to 600 °C at a rate of 10 °C/min in a nitrogen atmosphere. The samples were heated in aluminum trays to assess their temperature stability.

A DSC 3+ Star system from METTLER TOLEDO (Leicester, England) was used to analyze the material’s crystallization behavior between 10 and 260 °C at a rate of 10 °C/min in a N2 atmosphere. The endothermic peaks in the DSC thermograms were analyzed in order to examine the melting and crystallization properties of the HDPE samples. The heat of fusion ΔHm was calculated from the area under the melting peak, and percent crystallinity was calculated by comparing the sample heat of fusion with the heat of fusion of 100% crystalline sample (ΔHm_HDPE100%_ = 293 J/gm for HDPE). Using the data from the DSC thermogram and the following equation, the degree of crystallinity (Xc) was calculated.

Xc = (ΔHm/ΔHm_HDPE100%)_ × 100 (2)

The oxidation induction time (OIT) experiments consisted of heating the sample from room temperature to 200 °C in a nitrogen environment at a rate of 50 °C per minute. The temperature is then held at 200 °C for 5 min. Following that, the atmosphere in the chamber was changed to oxygen. Next, the time between the introduction of oxygen and the detection of the exothermic peak was noted [33].

## 3. Results and Discussion

### 3.1. Tensile Test Results

**Figure 2:**
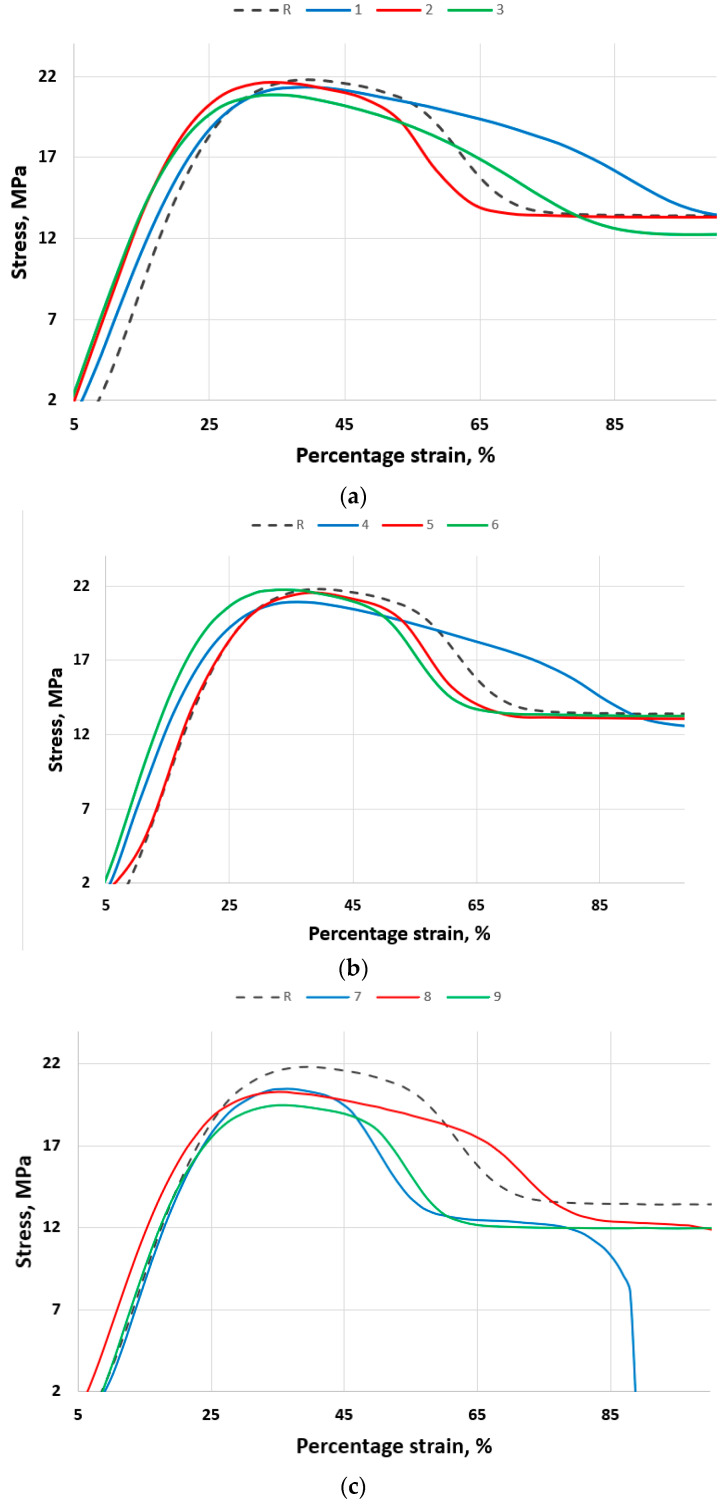
Stress–strain curves for different temperatures: (**a**) 20 °C; (**b**) 40 °C; (**c**) 60 °C.

The gradual leftward shift in the stress–strain curves (Figure 2) with increasing chlorine concentration indicates a reduction in the material’s ability to deform plastically. This behavior suggests that chlorine exposure leads to oxidative degradation and molecular chain scission, resulting in a stiffer, less ductile polymer. Consequently, the material fails at lower elongations, demonstrating the transition from a ductile to a brittle mechanical response, as shown in similar works [7,14].

At ambient temperature (≈20 °C), the chlorine-exposed HDPE specimens show only modest changes relative to the unexposed reference: the initial linear slopes are slightly steeper for exposed samples (a small increase in apparent stiffness), the peak stresses remain near the reference value (~22 MPa), and the post-yield (plastic) region is reduced progressively with chlorine concentration. The curves shift slightly left with increasing ppm, indicating a moderate loss of ductility; however, the material still exhibits significant plasticity before failure, particularly at low concentration (5–10 ppm).

At 40 °C, the same concentration series shows an amplified effect. All exposed samples have noticeably steeper initial slopes than at 20 °C (larger increase in apparent elastic modulus), while the peak stress values remain approximately similar to those at 20 °C but with sharper, more pronounced peaks. Most importantly, the post-yield region is markedly shortened, and the curves are shifted further left—especially for 10 and 20 ppm—indicating much earlier onset of plastic instability and substantially reduced elongation at break. The 20 ppm sample at 40 °C exhibits the strongest embrittlement: a steep stress drop after the peak and a low residual stress at high strain. Elevated temperature accelerates chlorine-induced embrittlement: stiffness increases modestly, but ductility and deformation capacity decrease substantially.

At 60 °C, the chlorine-exposed HDPE specimens exhibit a pronounced amplification of the degradation trends observed at lower temperatures. All exposed samples (5 ppm—sample 7, 10 ppm—sample 8, and 20 ppm—sample 9) show progressively greater embrittlement with increasing chlorine concentration.

The initial slopes of the stress–strain curves remain similar to the reference, indicating that stiffness is not substantially increased by exposure. However, the post-yield regions are sharply reduced, and the curves shift significantly leftward, showing premature plastic instability and loss of elongation at break. Sample 7 (5 ppm) maintains the highest tensile strength among the exposed series and preserves a limited capacity for plastic flow, while sample 9 (20 ppm) exhibits the strongest degradation effect: an abrupt stress drop immediately after the peak and minimal residual stress at high strain.

The material’s ability to undergo plastic deformation decreases substantially with chlorine concentration, while peak stress values remain roughly constant or slightly lower. Elevated temperature thus intensifies the oxidative and chain-scission mechanisms initiated by chlorine exposure, leading to a stiffer but more brittle behavior typical of thermo-oxidatively aged HDPE [34,35].

The quantitative tensile test results, for all exposure conditions, are summarized in Table 4.

### 3.2. XRD Results

**Figure 3:**
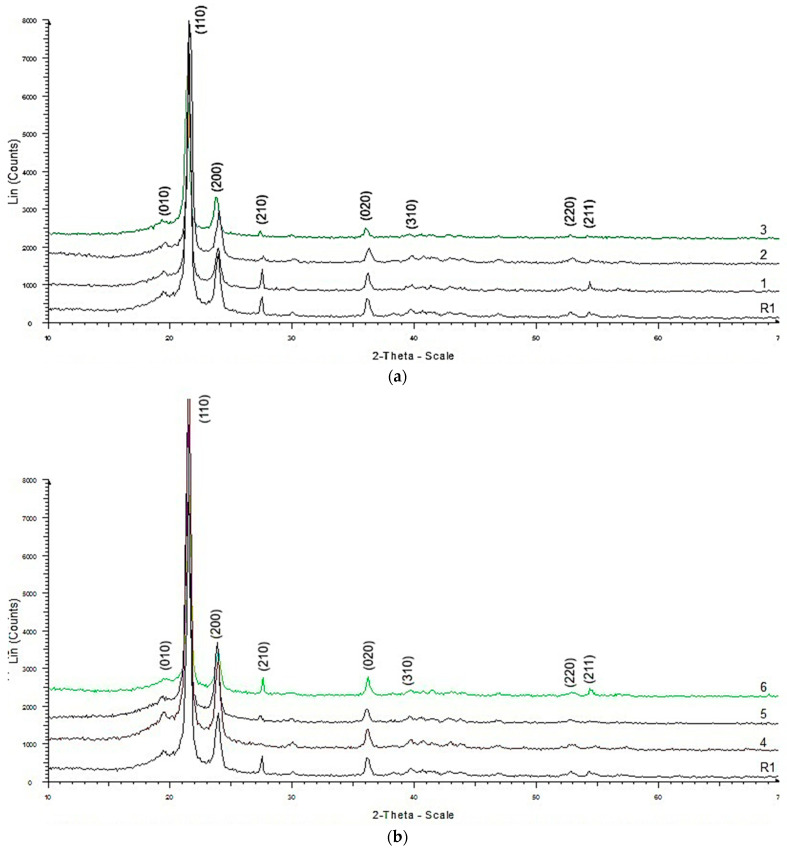

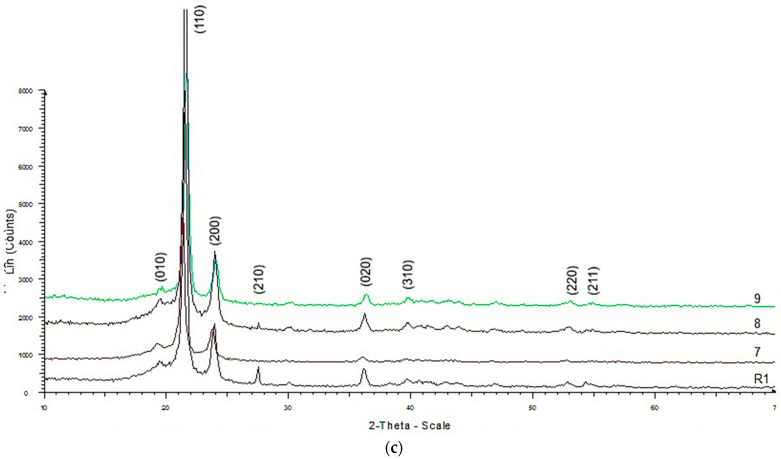
XRD spectra for HDPE samples exposed to different temperatures: (**a**) 20 °C; (**b**) 40 °C; (**c**) 60 °C.

The X-ray diffraction spectra exhibit a similar overall trend, with observable variations depending on the temperature and chlorine concentration to which the samples were exposed (Figure 3). Although the diffraction peaks in the 18–25° (2θ) range are intense and well defined, they do not reach the baseline, indicating the semi-crystalline nature of the polyethylene samples.

The main diffraction peaks identified in the XRD patterns (Figure 3) appear at 2θ = 21.47° (110), 23.88° (200), 27.47° (210), and 36.20° (020), corresponding to the orthorhombic crystal structure of polyethylene (space group Pnam (62)) according to ICDD card 00-054-1982. Additional minor peaks are observed at 2θ = 19.46° (010), 40.70° (310), 52.85° (220), and 54.35° (211), further confirming the orthorhombic polyethylene structure.

Based on the experimental data and an appropriate structural model, the crystalline lattice parameters of polyethylene were refined using the Rietveld method with the Diffracplus TOPAS 4.1 software. The refinement was carried out under the assumption that HDPE possesses an orthorhombic unit cell (space group Pnam (62)), in agreement with values reported in the literature by [36,37,38,39].

The calculated crystalline lattice parameters (a, b, and c) of the polyethylene samples fall within the ranges: a = 7.371–7.509 Å, b = 4.867–4.999 Å, and c = 2.506–2.683 Å (see Table 5), in agreement with values reported in the literature [36,37,38,39].

**Figure 4:**
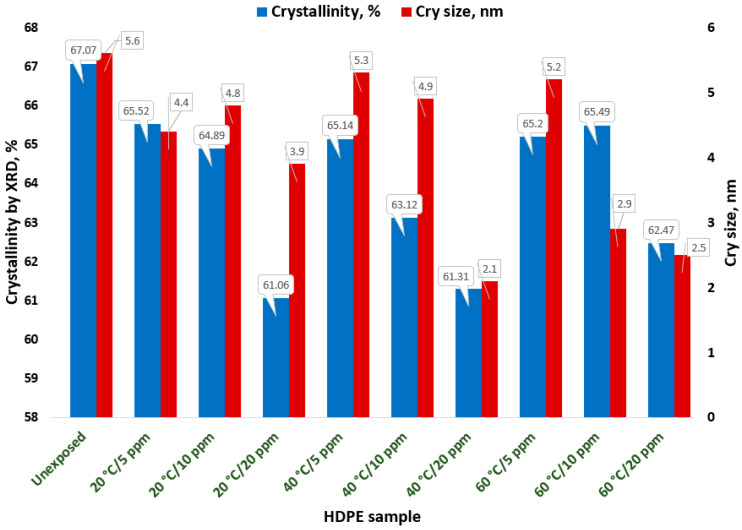
Crystallinity results obtained by XRD analysis.

The results of the XRD investigation are presented in Table 5 and Figure 4.

The calculated degree of crystallinity from XRD measurements is comparable to values reported for various HDPE types in previous studies [29,40,41].

The degree of crystallinity calculated from the XRD spectra, relative to the reference sample R, shows a decreasing trend with increasing temperature, and particularly with higher chlorine content. This reduction in crystallinity for samples exposed to varying temperatures and chlorine concentrations can be explained by the fact that XRD primarily probes the surface layers of the samples, which degrade over micron-scale depths due to interactions between the HDPE surface and progressively higher chlorine concentrations.

Surface degradation of HDPE exposed to solutions with high chlorine content has also been reported in previous studies [3]. The decrease in crystallite size with increasing exposure temperature of the HDPE sample surfaces indicates a reduction in molecular chain orientation, which correlates with the observed decrease in the degree of crystallinity.

**Figure 5:**
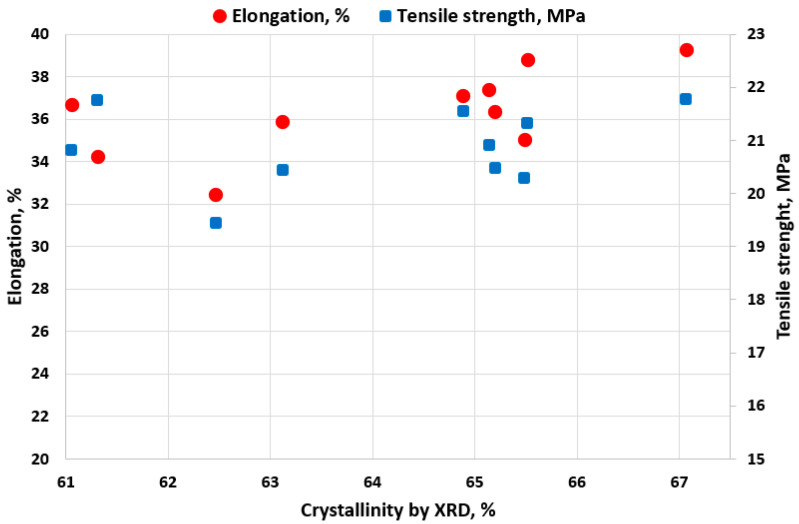
Correlation between crystallinity and mechanical performance of HDPE samples.

Figure 5 directly illustrates the impact of the varied exposure conditions on the material’s structural integrity and mechanical performance. The data clearly show that the samples shift away from the unexposed state (characterized by the highest crystallinity, approx 67.07%, and peak mechanical values, toward the lower left region of the plot, which represents degraded material (lower crystallinity and reduced mechanical properties). Specifically, the elongation at yield (red circles) demonstrates a clear positive relationship with crystallinity; as degradation progresses and crystallinity decreases, the material’s ductility is consistently reduced. Conversely, the tensile strength (blue squares) exhibits a more complex and scattered relationship with crystallinity. While degradation is evident across all exposed samples, the final yield strength appears less dependent on the bulk change in crystallinity. Conversely, the tensile strength (blue squares) exhibits minimal change during the exposure conditions, remaining tightly clustered along the Y-axis (ranging from approximately 19.44 MPa to 21.77 MPa). This minimal change indicates that while degradation is evident across all exposed samples (as confirmed by the reduction in crystallinity and elongation), the final yield strength appears less dependent on the bulk change in crystallinity.

Previous experimental studies [3,5,42,43] reported the degradation of the HDPE surface and the modification of molecular structures with aging after exposure to solutions with high chlorine concentrations. The crystallinity degree values calculated by the Rietveld method (Table 5) reported to the control sample R showed a decreasing trend with increasing temperature and especially with increasing chlorine content. The decrease in crystallinity of the samples may be due to structural changes occurring in the HDPE samples through the relocation and concentration of amorphous structures, on the one hand correlated with chain breakage and reduction in interlamellar thickness, on the other hand, in agreement with previous research [5,42,44,45].

### 3.3. FTIR Analysis Results

**Figure 6:**
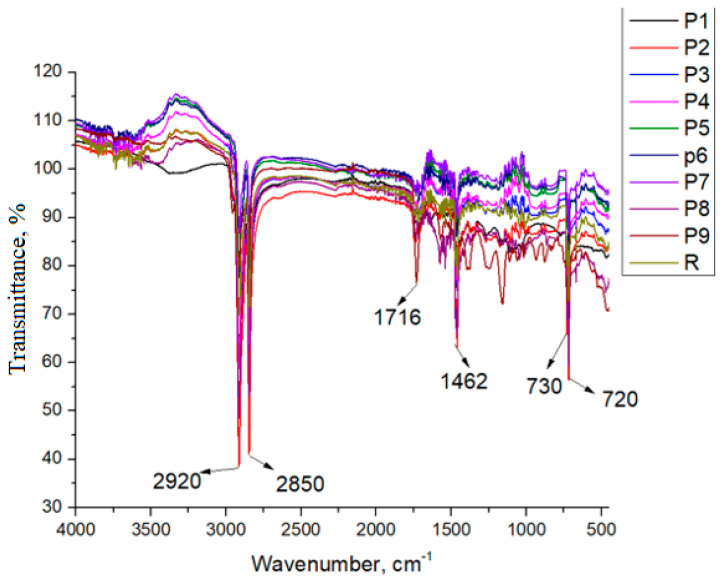
FTIR spectra of HDPE magnification of the 4000–400 cm^−1^ region.

As can be seen in Figure 6, all of the samples show peaks specific to the high-density polyethylene. The intense absorption bands at approximately 2920 and 2850 cm^−1^, corresponding to the asymmetric and symmetric stretching modes of CH_2_ groups, confirm the polyethylene backbone and indicate that the bulk polymer structure remains predominantly unchanged after processing. The deformation modes observed in the 1472–1462 cm^−1^ region provide additional information regarding chain packing and crystallinity, parameters commonly used to evaluate structural organization in HDPE [46].

**Figure 7:**
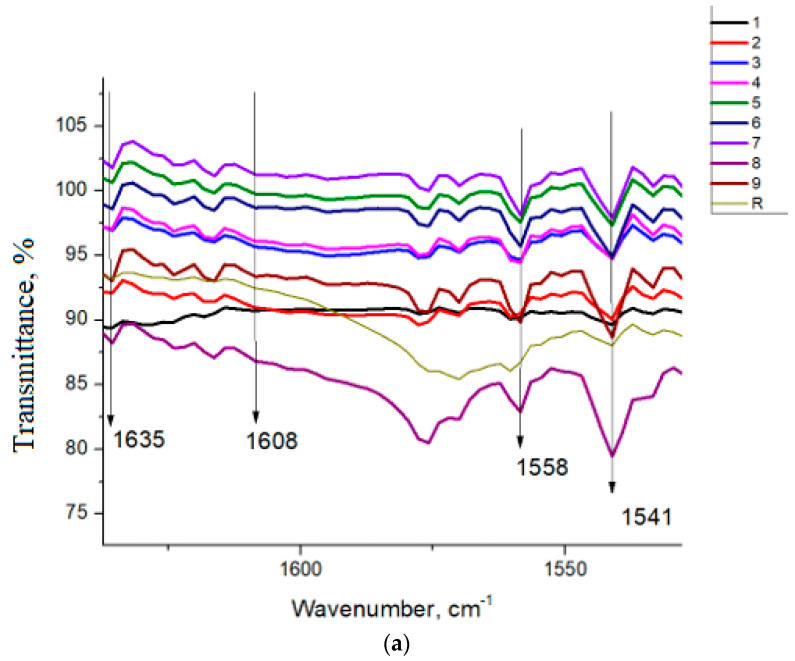

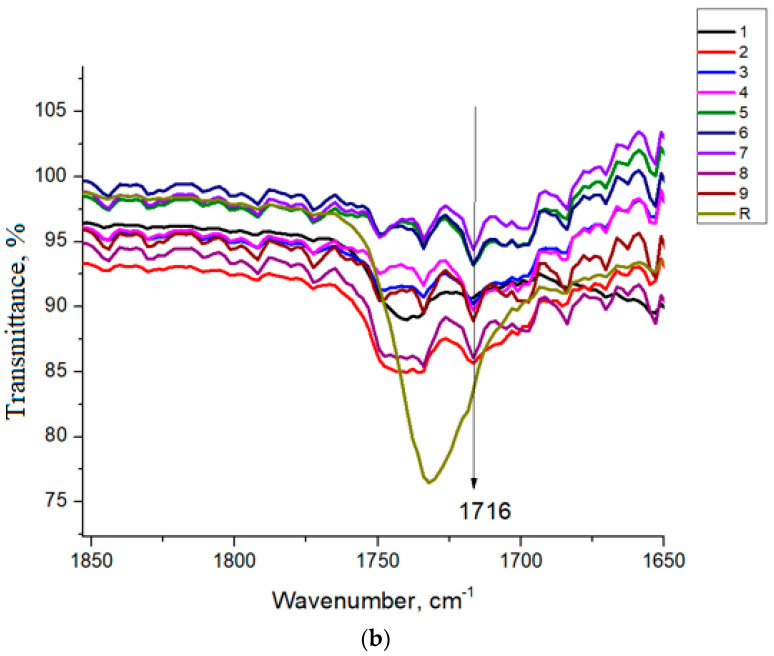
FTIR spectra of HDPE: (**a**) magnification of 1650–1500 cm^−1^ region, (**b**) magnification of 1850–1650 cm^−1^ region.

The presence of CH_2_ rocking bands at 730 and 720 cm^−1^ indicates a semicrystalline phase typical of HDPE, consistent with both virgin and recycled grades. Moderate variations detected in the 1300–900 cm^−1^ region are in agreement with previously reported spectra for recycled polyethylene and may be attributed to minor residual impurities originating from the recycling stream [47,48]. Evidence of chemical treatment–induced oxidation becomes apparent upon examination of the carbonyl region. A progressive increase in the band intensity at 1716 cm^−1^ was observed across all samples, accompanied by the appearance of new features in the 1608–1635 cm^−1^ range. These bands are consistent with the formation of carboxyl-containing oxidation products and indicate that the applied treatment promotes chain scission and subsequent formation of oxygenated groups [5,49] (Figure 7). The appearance of C–O stretching absorptions in the 1100–1000 cm^−1^ interval indicates the formation of oxygenated degradation products, reinforcing the presence of secondary oxidation reactions typical of late-stage deterioration.

The observed oxidation pathways align with previously reported degradation mechanisms for HDPE exposed to oxidizing disinfectants. Khan et al. [50] demonstrated that immersion of HDPE materials in NaOCl and ClO_2_ solutions leads to a significant increase in carbonyl index, reflecting the conversion of intermediate ketone/aldehyde groups into carboxylates during oxidative aging. Moreover, exposure to NaOCl has been shown to induce not only oxidative reactions but also surface chlorination, with degradation kinetics strongly influenced by solution concentration, pH, temperature, and exposure time [51].

Additional support for these mechanisms is provided by Bottone et al. [52] who reported that NaOCl activates both aliphatic and aromatic carbon sites, facilitating the formation of carbonyl and carboxylate species as confirmed by FTIR and XPS analyses. These findings corroborate the transformations observed in the present study and highlight the susceptibility of HDPE to oxidative–chlorinative degradation under chemical treatment.

### 3.4. Thermal Analysis Results

#### 3.4.1. TGA Analysis Results

**Figure 8:**
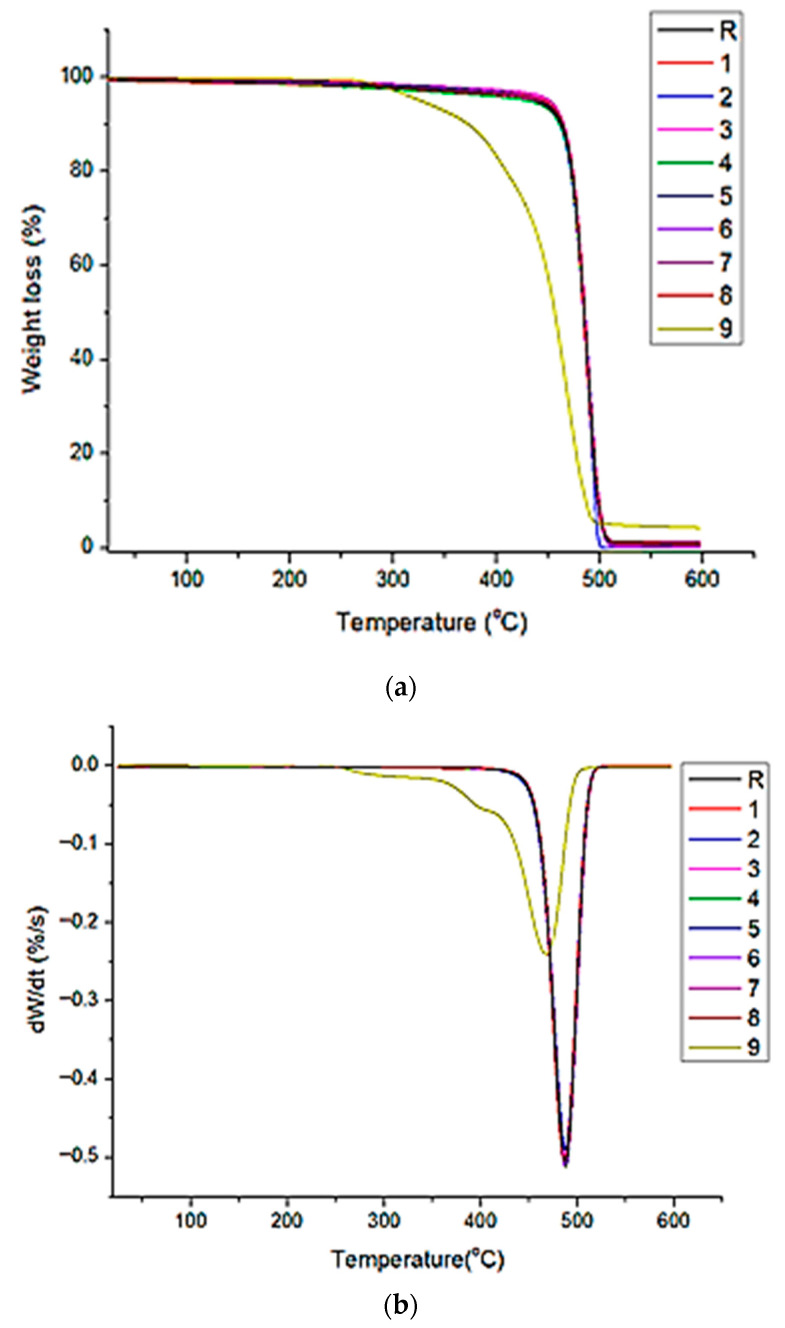
(**a**) TGA and (**b**) DTG profiles of the HDPE samples.

To evaluate the decomposition of HDPE samples, the TGA profiles were analyzed at a constant heating rate of 10 °C/min, under nitrogen, and displayed in Figure 8. As can be seen in Figure 8, all the samples except sample 9 exhibited a single thermal degradation step with a total weight loss between 94% and 96%, which starts at 430 °C and ends around 510 °C, caused by chain scission in the polymer and the quick evaporation of carbon monoxide that results from the process. Similar HDPE degradation profiles are reported in the literature under inert conditions [53].

The values of maximum degradation temperature (Td), T5%, T50%, and T95% are obtained from the peak of the DTG curve and shown in Table 6. The data indicate a shift in T5%, T50%, and T95% values towards lower temperatures, which suggests an increased degradation rate of the samples. For example, the T5% temperatures decrease from 449 °C for sample 1 (20 °C and chlorine concentration of 5 ppm) to 326 °C for sample 9 (60 °C and chlorine concentration of 20 ppm). The observed trend is possibly caused by changes in the polymer chain structure of the samples, as supported by the FT-IR analysis. This phenomenon is better observed for sample 9, which was subjected to high temperature (60 °C) and chlorine concentration (20 ppm), and which shows an additional peak degradation with a temperature maximum at 398 °C and a mass loss of 14.12%. These findings are in agreement with Amelia Habas-Ulloa et al. [54] which affirms that the thermal stability of HDPE polymer is directly correlated with its average molecular weight, leading to diminished thermal properties at lower molecular weights.

#### 3.4.2. DSC Analysis Results

DSC analysis measures changes in crystallization and melting behavior resulting from chemical structure modification during degradation [55].

**Figure 9:**
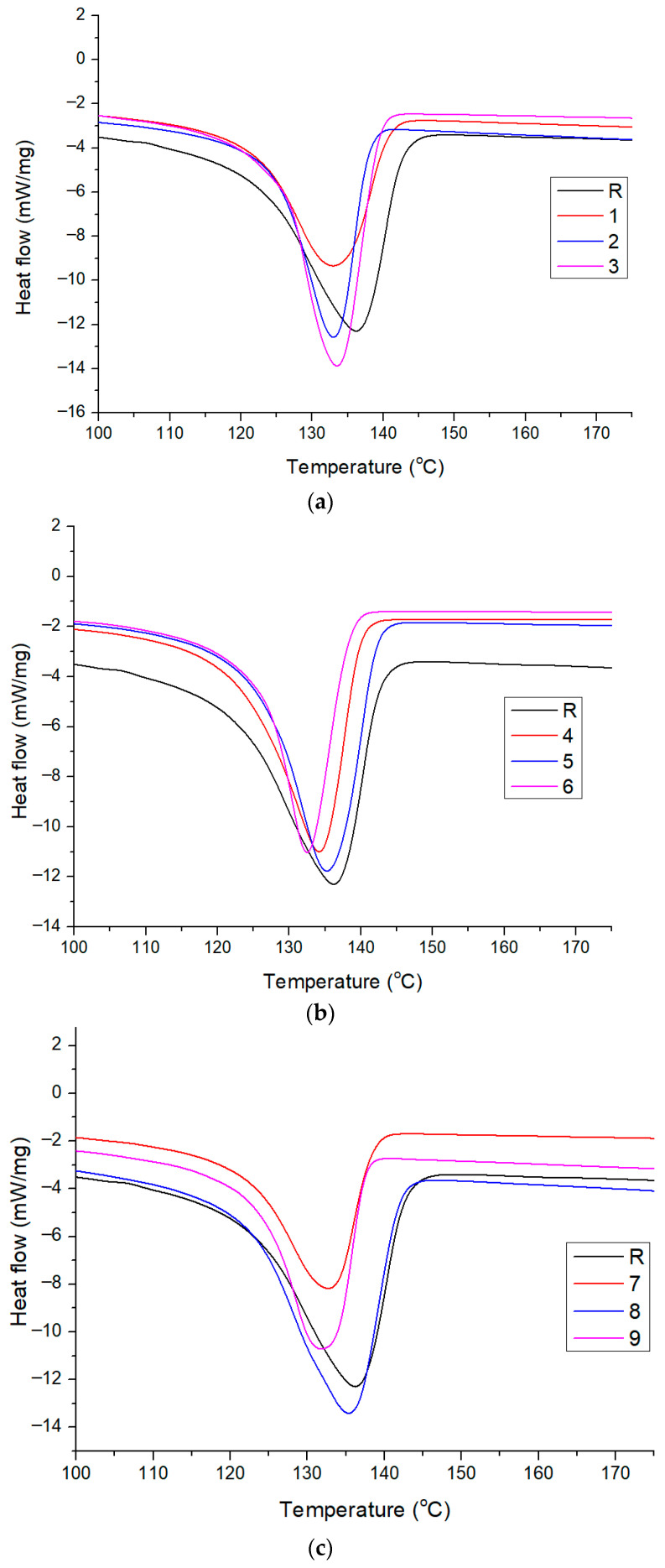
DSC thermograms (melting peak) of the HDPE samples exposed to different temperatures: (**a**) 20 °C; (**b**) 40 °C; (**c**) 60 °C.

As can be seen in Figure 9, there is a shift in the melting temperature depending on the tested conditions. For example, when the influence of chlorine was studied, at a constant temperature of 20 °C, a decrease in Tm is observed from 136.32 °C (sample 1) to 133.40 °C (sample 2) and 132.78 °C (sample 3), respectively, probably caused by polymer surface chain degradation leading to the formation of structures with a lower melting point [56], which causes a slight shift in the endothermic peak. The same behavior was noticed for the constant temperatures of 40 °C and 60 °C. A different behavior in which Tm increases slightly with almost one degree and 0.28 °C., after which it decreases with almost 1.18 °C, and 1.33 °C, respectively, was observed when the influence of chlorine concentration of 10 and 20 ppm was studied. This fact may be caused by the thermo-oxidative degradation reaction, which leads to chemical aging, causing the appearance of carbonyl groups in the FT-IR spectra (Figure 7). Compared with the R (standard) sample, the Tm shift is more noticeable for the sample 9 treated under maximum conditions.

**Figure 10:**
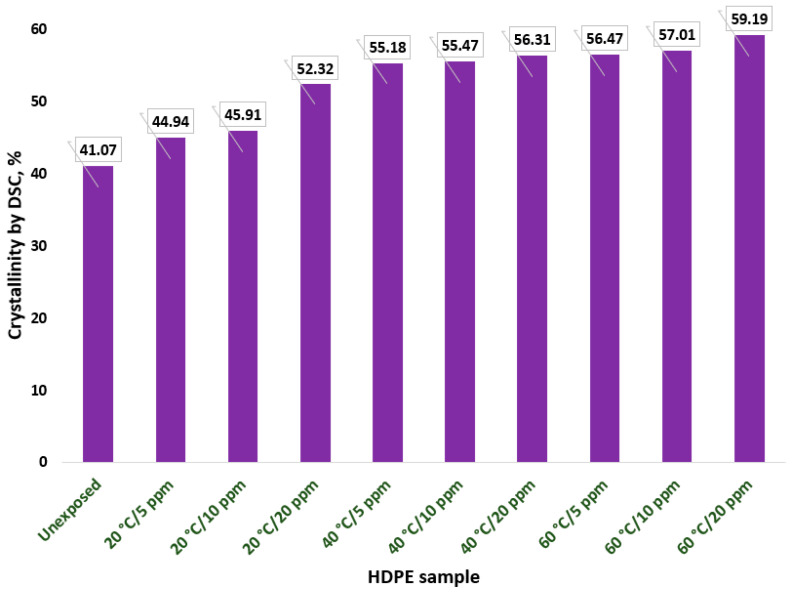
Crystallinity results obtained by DSC analysis.

The degree of crystallinity, calculated from the ΔHm values obtained from the DSC curves (Figure 9), is presented in Table 6 and Figure 10 and indicates an increase in the degree of crystallinity for all samples from 44.94% (sample 1) to 59% (sample 9). To note that the highest values of 56.47%, 57.01%, and 59.19% were determined for samples 7 to 9. This behavior can be explained by the fact that the degree of crystallinity in HDPE can increase under chlorine aging because the chlorine-induced oxidation, which primarily degrades the less-ordered amorphous regions of the polymer, breaks down the amorphous chains and leaves the more stable crystalline regions intact. This process leads to a relative increase in the proportion of crystalline material as the amorphous component is consumed and fragmented, which can make the material appear more brittle. Additionally, an increase in crystallinity may serve as an indicator of polyethylene degradation through chain scissions, which occur primarily in the amorphous phase. As a result, entanglements in the amorphous phase are reduced, allowing molecules to form crystals due to their higher mobility. The chemical degradation of molecules eventually leads to highly crystallizable, short chains. Therefore, the more chemical degradation progresses, the higher proportion of short chains will crystallize [57].

#### 3.4.3. Antioxidant Loss

Degradation of polymers is primarily influenced by oxidation reactions involving free radicals. Researchers indicate that HDPE pipe degradation caused by long-term exposure to water containing free available chlorine occurs in three main stages: (1) chlorinated water attacks the pipe surface, resulting in signatures of oxygen, chlorine, hydroxyl, and vinyl components; (2) degradation breaks interlamellar tie molecules, increasing chain layering, crystalline content, and molar mass distribution; and (3) subsequent oxidation leads to molecular weight reduction due to chain scission, making the pipe brittle [2]. Therefore, to enhance the stability of HDPE by retard or halting the oxidation process, antioxidant packages with a combination of two or more types of compounds are incorporated into pipes [58]. Each unique antioxidant formulation may be tailored to specific applications and manufacturers’ requirements. A prominent group of antioxidants utilized in the industry includes hindered phenolic antioxidants (Irganox 1010, 1330, 1024, and 3114), which are noted for their pollution-free and non-discoloring properties, contrasting with the toxicity and discoloration often associated with amine antioxidants [59].

**Figure 11:**
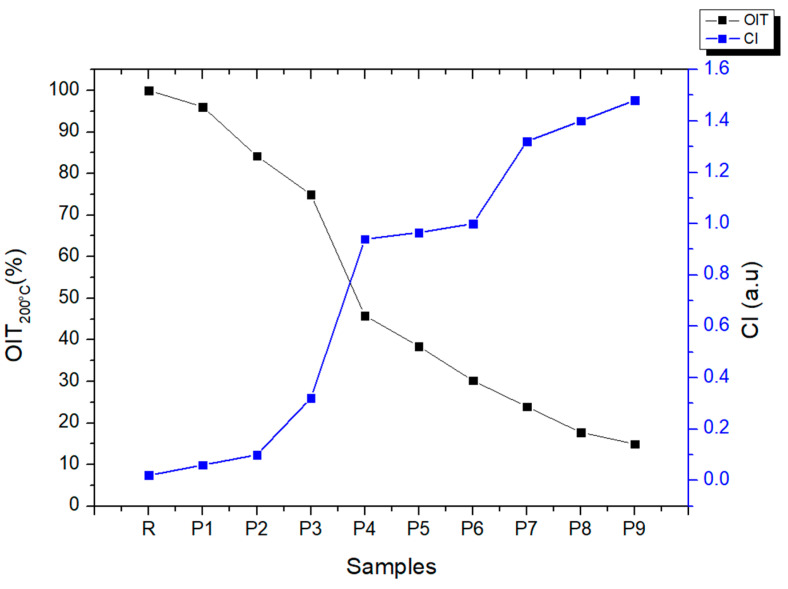
Effect of aging conditions on OIT percent and carbonyl index.

To study the impact of disinfectant exposure on the HDPE samples at different temperatures and chlorine concentrations, the oxidative induction time method (OIT) was used [33]. OIT measurements cannot specify antioxidant types but can assess the relative concentration of antioxidants in aging specimens. Since OIT is proportional to antioxidant concentration, we considered the OIT value of the untreated sample of 66.85 min to represent a 100% antioxidant concentration [58]. As seen in Figure 11, all the samples showed that the rate of antioxidant depletion depends on the temperature and the concentration of chlorine in the water. For HDPE samples aged under mild conditions of 20 °C, a slow oxidation was observed, with the OIT value decreasing from 96.05% (sample 1) to 84.21% for sample 3, which could be due to the change in the molar mass distribution due to chain breakage caused by the attack of chlorinated water. A greater reduction in OIT was observed at a temperature of 40 °C, where the OIT decreases to 74.97%, 45.85%, and 38.44% for samples 3, 4, and 5. However, the samples met the OIT minimum of 20 min according to ASTM D3895-19 [60]. The most intense OIT reduction occurred for the HDPE pipes solution aged at 60 °C. The percentage loss of OIT was of 23.93% 17.74% and 14.95%. The simultaneous penetration of water into HDPE, the increased migration of antioxidants from the polymer, and potentially the heat degradation of the antioxidant from the polymer could all be responsible for this quick reduction [2]. This statement is supported by the carbonyl index (CI), which increases as the aging conditions become more severe (Figure 11), from 0.06 (sample 1) to 1.48 (sample 9). This trend is attributed to the formation of carbonyl groups—such as ketones, esters, carboxylic acids, and aldehydes—resulting from oxidation reactions that occur due to antioxidant loss.

### 3.5. Statistical Analysis Results

**Figure 12:**
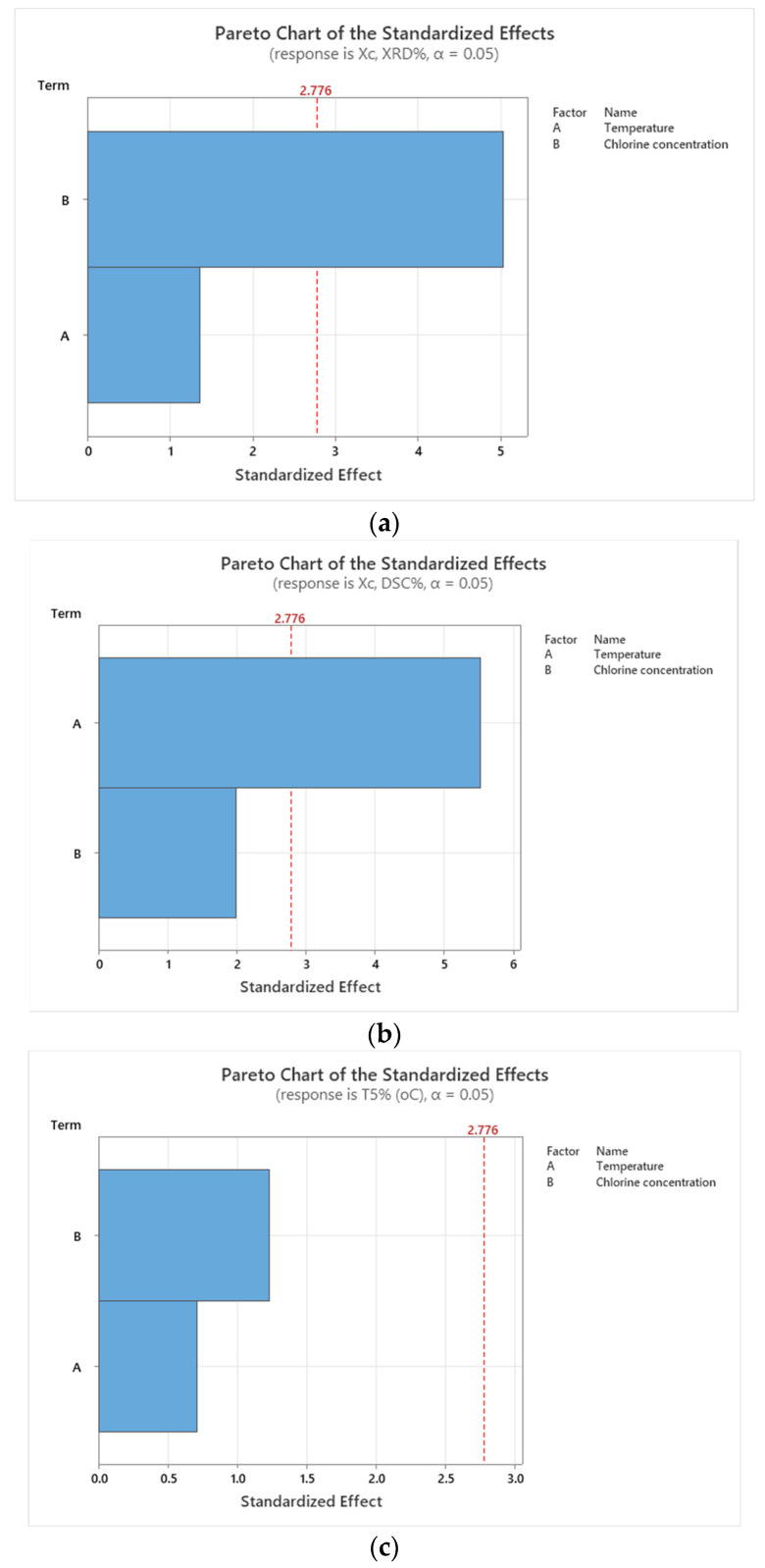
Pareto charts for: (**a**) Crystallinity (XRD); (**b**) Crystallinity (DSC); (**c**) T5%.

Figure 12 shows that temperature and chlorine concentration influence HDPE degradation differently depending on the measured response. For XRD crystallinity (Figure 12a), chlorine concentration is the dominant factor, clearly surpassing the significance threshold, while temperature has only a minor effect. This indicates that surface structural degradation is driven mainly by chlorine-induced oxidation. In contrast, DSC crystallinity (Figure 12b) is governed primarily by temperature, which significantly exceeds the threshold, whereas chlorine concentration has a smaller, non-significant influence; this reflects that bulk crystallinity is controlled mainly by thermally driven molecular reorganization rather than chemical attack. For T5% (Figure 12c), chlorine concentration again emerges as the most influential factor, confirming that early thermal decomposition and molecular weight loss are primarily associated with oxidative chain scission caused by chlorine exposure.

**Figure 13:**
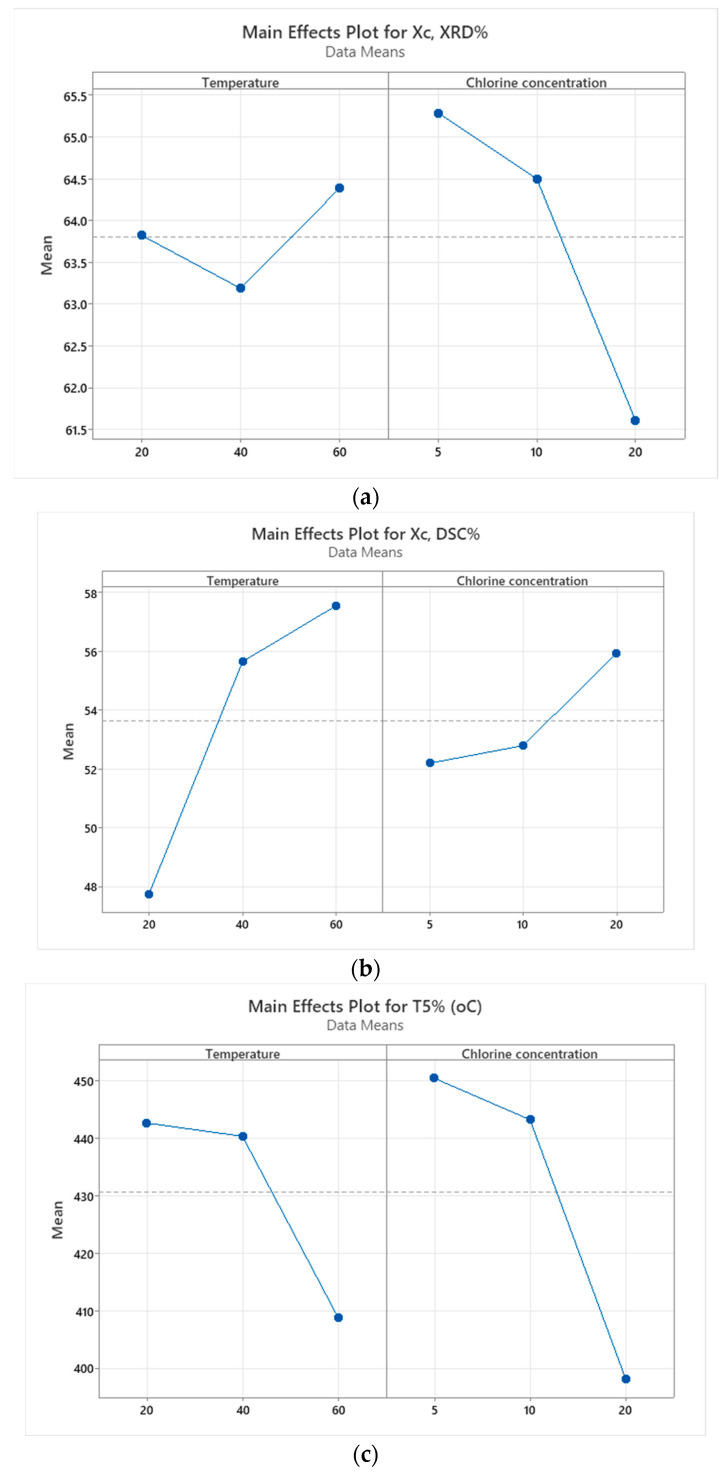
Main effect plots for: (**a**) Crystallinity (XRD); (**b**) Crystallinity (DSC); (**c**) T5%.

Figure 13 illustrates how temperature and chlorine concentration influence the crystallinity and thermal stability of HDPE. For XRD crystallinity (Figure 13a), chlorine concentration has a clear decreasing trend, with crystallinity dropping sharply at 20 ppm, while temperature shows only modest variation. This confirms that surface crystallinity is primarily reduced by increasing chlorine levels. In contrast, DSC crystallinity (Figure 13b) increases strongly with temperature, reflecting thermally driven chain mobility and recrystallization, while chlorine concentration shows only minor changes. For T5% (Figure 13c), both temperature and chlorine concentration decrease thermal stability, but the most pronounced drop occurs at 60 °C and higher chlorine levels, indicating that oxidative degradation, amplified by heat, is the key driver of early mass loss.

The results from Table 7 show that for XRD-based crystallinity, chlorine concentration is the dominant factor, explaining more than 84% of the variation and exhibiting a significant effect, while temperature plays only a minor and non-significant role. In contrast, crystallinity measured by DSC is controlled mainly by temperature, which accounts for over 80% of the variability and is statistically significant, whereas chlorine concentration has a weaker and non-significant influence. For T5%, neither factor is individually significant; however, the variability is largely governed by their combined interaction, which contributes nearly half of the total variation.

**Figure 14:**
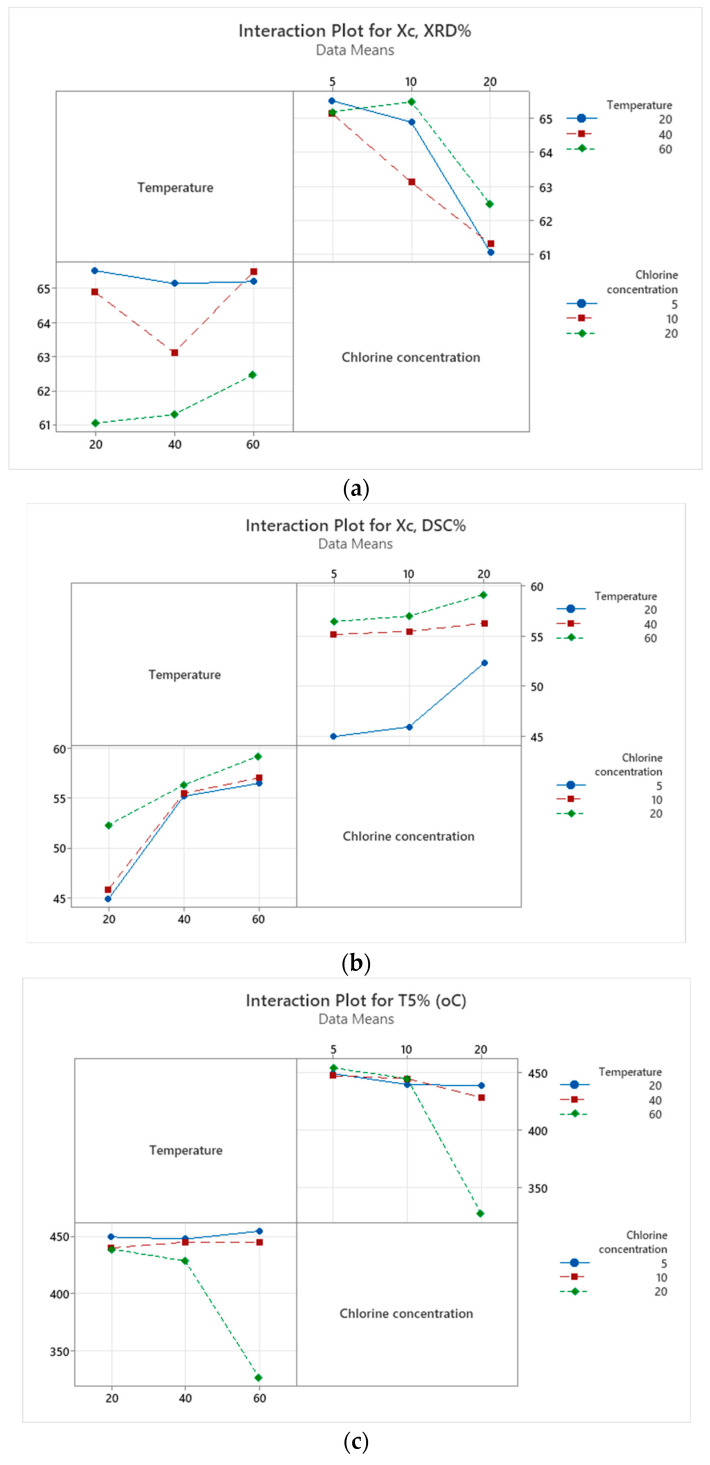
Interaction plots for: (**a**) Crystallinity (XRD); (**b**) Crystallinity (DSC); (**c**) T5%.

Figure 14 shows that temperature and chlorine concentration interact differently for the three measured properties. XRD determined that crystalline is relatively stable at low chlorine levels, but at 20 ppm chlorine, it decreases sharply with increasing temperature, indicating a negative interaction between high temperature and high chlorine content. In contrast to XRD, crystallinity measured by DSC increases with both temperature and chlorine concentration. Thermal stability is almost unaffected at 5–10 ppm chlorine, but at 20 ppm chlorine, a strong drop in T5% occurs at high temperature, demonstrating a detrimental interaction between high chlorine content and high processing temperature on thermal stability.

The shapes and orientations of the interaction curves further support these conclusions. Nearly horizontal lines indicate that a factor has little effect on the response, whereas steeper, more vertical lines show a dominant influence of that factor. Parallel lines suggest that the factors act independently, while intersecting or diverging lines reveal a significant interaction between temperature and chlorine concentration. In the present case, the strong curvature and crossing of the lines at 20 ppm highlight that the response is not governed by a single factor but by their combined effect.

Therefore, low–moderate chlorine produces mild, predictable trends, while 20 ppm chlorine causes strong interactions, reducing XRD crystallinity and thermal stability at high temperatures, but increasing the DSC-measured crystallizable fraction.

Quantitative differences visible in the interaction plots further reinforce these findings. For XRD crystallinity (Figure 14a), values remain clustered around 64–65% at 5–10 ppm chlorine for all temperatures, but at 20 ppm chlorine, the crystallinity drops markedly from approximately 63% at 60 °C to about 61% at 20 °C. In contrast, DSC-measured crystallinity (Figure 14b) shows a consistent increase with both factors: at 20 ppm chlorine, the crystallinity rises from roughly 53% at 20 °C to nearly 59% at 60 °C, confirming that higher chlorine enhances the fraction of material that recrystallizes upon heating. Regarding thermal stability, T_5_%, it can be observed from Figure 14c that across chlorine concentrations of 5 ppm and 10 ppm, the T5% values remain relatively stable as temperature increases. The observed variation is minor, within approximately 2–5 °C, and all values stay within the range of 448–455 °C. This indicates that in low- to moderate-chlorine environments, temperature alone does not substantially influence the onset of thermal degradation. However, at 20 ppm chlorine concentration, the effect of temperature becomes more pronounced. T5% decreases moderately from ~445 °C at 20 °C to ~430–435 °C at 40 °C, followed by a sharp decline to approximately 330 °C at 60 °C. This indicates that elevated temperatures significantly amplify chlorine-induced thermal degradation when chlorine concentration reaches critical levels.

## 4. Conclusions

The main conclusions drawn from this study are as follows:

- HDPE undergoes significant chemical, structural, thermal, and mechanical degradation in chlorinated aqueous environments, with deterioration strongly accelerated at elevated temperatures.
- Mechanical performance deteriorates sharply, with tensile tests showing a transition from ductile to brittle behavior. Samples aged at 60 °C and 20 ppm chlorine exhibited almost complete loss of post-yield deformation and premature failure, despite peak stresses remaining close to the reference (~22 MPa).
- Structural degradation was confirmed by XRD, with crystallinity decreasing from 67.07% (reference) to ~61% at high chlorine levels, and crystallite size shrinking from 5.60 nm to 2.10–2.50 nm, indicating loss of molecular ordering in surface layers.
- Chemical changes identified by FTIR revealed progressive oxidation, including a pronounced increase in the carbonyl band (1716 cm^−1^) and the formation of additional oxygenated species, consistent with chlorine-induced chain scission.
- Thermal stability decreased substantially, as TGA showed T5% dropping from 450 °C (reference) to 326.99 °C under the most aggressive aging conditions, along with the appearance of a secondary degradation peak at 398 °C.
- DSC analysis indicated reductions in melting temperature (from 136.32 °C to 131.67 °C) and crystallinity changes up to 59.19%, reflecting the breakdown of amorphous regions and recrystallization of shorter chain fragments.
- Collectively, the results demonstrate that chlorine aging drives coupled thermo-oxidative and structural degradation mechanisms that reduce HDPE’s toughness, accelerate embrittlement, and diminish its ability to withstand mechanical loads.
- The pronounced loss of thermal stability, increased oxidation, and reduced structural order highlight the vulnerability of HDPE components in chlorinated water systems, especially at high temperatures, underscoring the need for careful control of disinfectant concentration and operating temperature.
- These findings support the development of improved lifetime prediction models for HDPE infrastructure exposed to chlorinated environments.

### Limitations and Future Work

The study’s 45-day exposure period and static immersion conditions do not capture the full complexity of real distribution networks, including internal pressure, cyclic loading, and variable disinfectant levels.

Only free chlorine (NaOCl) was evaluated, whereas actual systems may involve mixtures of disinfectants, by-products, and pH variations that alter degradation kinetics.

Future work should incorporate long-term aging, mechanical loading, and dynamic hydraulic conditions to develop more robust and realistic lifetime prediction models for HDPE piping systems.

## Figures and Tables

**Table 1:** Parameters and corresponding levels utilized in DOE analysis.

Parameter	Level		
	1	2	3
Temperature, °C	20	40	60
Chlorine concentration, ppm	5	10	20

**Table 2:** Experimental conditions.

Exp. No.	Temperature, °C	Chlorine Concentration, ppm
1	20	5
2	20	10
3	20	20
4	40	5
5	40	10
6	40	20
7	60	5
8	60	10
9	60	20

**Table 3:** Fundamental material parameters of the HDPE samples.

Property	Value	Unit	Test Method
Melt Index	0.3	g/10 min	ASTM D1238 [23] @190 °C, 5 kg
Melt Index	0.8	g/10 min	ASTM D1238 [23] @190 °C, 21.6 kg
Density	0.961	g/cm^3^	ASTM D792 [24]
Durometer Hardness	63	Shore D	ASTM D2240 [25]
Degree of crystallinity [26]	60 … 80	%	

**Table 4:** Summary of tensile test results.

Specimen	Tensile Strength at Yield, MPa	Elongation at Yield, %
Unexposed (R)	21.77	39.28
1	21.33	38.78
2	21.54	37.09
3	20.82	36.68
4	20.9	37.38
5	20.44	35.85
6	21.76	34.24
7	20.48	36.36
8	20.29	35.04
9	19.44	32.45

**Table 5:** Lattice parameters of polyethylene (HDPE) space group Pnam (62).

Sample	a (Å)	b (Å)	c (Å)	Cry Size (nm)	*X_c,XRD_* %
R	7.409	4.949	2.650	5.60	67.07
1	7.404	4.957	2.544	4.40	65.52
2	7.390	4.997	2.529	4.80	64.89
3	7.408	4.982	2.506	3.90	61.06
4	7.385	4.999	2.513	5.30	65.14
5	7.401	4.974	2.517	4.90	63.12
6	7.509	4.896	2.610	2.10	61.31
7	7.391	4.959	2.591	5.20	65.20
8	7.415	4.931	2.611	2.90	65.49
9	7.429	4.867	2.683	2.50	62.47

**Table 6:** The temperatures corresponding to mass loss of 5%, 50%, and 95% (T5%, T50%, and T95%) and the maximum degradation temperature (Td).

Sample	Maximium Degradation Temperature (Td) (°C)	T5% (°C)	T50% (°C)	T95% (°C)	T_m_ (°C)	X_c_ (%)
R	489	450.10	486.3	501.8	136.45	41.07
1	487.33	449.28	485.4	500.93	136.32	44.94
2	487.00	439.78	485.42	500.9	133.40	45.91
3	487.00	438.80	484.54	499.12	132.78	52.32
4	488.50	447.53	486.3	502.43	134.52	55.18
5	487.17	444.90	485.4	501.1	134.34	55.47
6	487.00	428.51	484.54	500.9	133.00	56.31
7	487.33	454.40	485.42	502.5	133.78	56.47
8	486.50	444.90	484.54	501.8	133.16	57.01
9	469.17	326.99	456.89	491.66	131.67	59.19

**Table 7:** Statistical parameters.

Response	R^2^	Factor	F-Value	*p*-Value	Contribution
*X_c_* (XRD)	92.13%	Temperature	2.05	0.244	8.06%
		Chlorine concentration	21.26	0.007	84.07%
*X_c_* (DSC)	93.63%	Temperature	25.86	0.005	81.52%
		Chlorine concentration	3.81	0.119	12.11%
T5%	100%	Temperature	0.78	0.519	17.17%
		Chlorine concentration	1.74	0.286	38.56%
		2-way interaction Temperature Chlorine concentration	-	-	44.26%

## Data Availability

The original contributions presented in this study are included in the article. Further inquiries can be directed to the corresponding author.
